# Effects of Benzo[k]fluoranthene at Two Temperatures on Viability, Structure, and Detoxification-Related Genes in Rainbow Trout RTL-W1 Cell Spheroids

**DOI:** 10.3390/toxics13040302

**Published:** 2025-04-12

**Authors:** Telma Esteves, Fernanda Malhão, Eduardo Rocha, Célia Lopes

**Affiliations:** 1 Laboratory of Histology and Embryology, Department of Microscopy, ICBAS—School of Medicine and Biomedical Sciences, University of Porto, Rua Jorge Viterbo Ferreira 228, 4050-313 Porto, Portugal; up201805154@edu.fc.up.pt (T.E.); fcmalhao@icbas.up.pt (F.M.); cclopes@icbas.up.pt (C.L.); 2 Group of Animal Morphology and Toxicology, Interdisciplinary Centre of Marine and Environmental Research (CIIMAR/CIMAR), University of Porto, Terminal de Cruzeiros do Porto de Leixões, Av. General Norton de Matos s/n, 4450-208 Matosinhos, Portugal

**Keywords:** benzo(k)fluoranthene, PAHs, global warming, in vitro models, spheroids

## Abstract

Polycyclic aromatic hydrocarbons (PAHs) and global warming impact aquatic ecosystems, eventually interacting. Monolayer (2D) cultures of cell lines, such as the rainbow trout liver RTL-W1, are employed for unveiling toxicological effects in fish. Nonetheless, three-dimensional (3D) models constitute an alternate paradigm, better emulating in vivo responses. Here, ultra-low attachment (ULA) plates were used to generate ten-day-old RTL-W1 spheroids for exposure to a control, a solvent control (0.1% DMSO) and the model PAH benzo[k]fluoranthene (BkF) at 10 and 100 nM and at 18 and 23 °C (thermal stress). After a 4-day exposure, spheroids were analyzed for viability (alamarBlue and lactate dehydrogenase), biometry (area, diameter and sphericity), histocytology (optical and electron microscopy), and mRNA levels of the detoxification-related genes cytochrome P450 (*CYP*)*1A*, *CYP3A27*, aryl hydrocarbon receptor (*AhR*), glutathione S-transferase (*GST*), uridine diphosphate–glucuronosyltransferase (*UGT*), catalase (*CAT*), multidrug resistance-associated protein 2 (*MRP2*) and bile salt export protein (*BSEP*). Immunocytochemistry (ICC) was used to assess CYP1A protein expression. Neither temperature nor BkF exposure altered the spheroids’ viability or biometry. BkF modified the cell’s ultrastructure. The expression of *CYP1A* was augmented with both BkF concentrations, while *AhR*’s increased at the higher concentration. The CYP1A protein showed a dose-dependent increase. Temperature and BkF concurrently modelled *UGT*’s expression, which increased in the 100 nM condition at 23 °C. Conversely, *CYP3A27*, *MRP2*, and *BSEP* expressions lowered at 23 °C. *CAT* and *GST* mRNA levels were uninfluenced by either stressor. Overall, BkF and temperature impacted independently or interactively in RTL-W1 spheroids. These seem to be useful novel tools for studying the liver-related effects of temperature and PAHs.

## 1. Introduction

Since the 19th century, the average Earth global temperature has increased by 1.2 °C [1], and projections indicate that an increase of 1.5 °C could be reached by 2030 [2]. The average global temperature is nearing 1.5 °C above pre-industrial levels, raising concerns about surpassing the 1.5 °C threshold established by the Paris Agreement [3,4]. Due to climate change, heatwaves are expected to become more frequent [5], and as water temperatures rise because of increases in air temperatures, fluvial habitats may be especially affected [6]. In freshwater ecosystems, warmer temperatures impact aquatic life by causing stress, reducing reproduction rates and increasing mortality [7]. Cold-water species, such as trout, are particularly vulnerable, as higher water temperatures may reduce flow rates and force them to migrate to cooler areas, interfering with life cycle events [8]. Furthermore, abiotic stressors such as temperature also impact liver metabolization, leading to the induction of antioxidant responses and increased levels of heat shock proteins [9,10].

Heatwaves can also lead to extensive forest fires, introducing polycyclic aromatic hydrocarbons (PAHs) into the environment. Evidence suggests that terrestrial inputs to water following fires contribute to the increase in PAH levels in lakes and rivers [11] and distress aquatic animals [12]. Anthropogenic sources, such as urban and agricultural runoffs, further contribute to the prevalence of PAHs in aquatic environments [13]. Studies have shown that, among others, PAHs in fish impact bone development [14], disrupt early development [15], and promote carcinogenesis [13]. In the fish liver, PAH exposure has been linked to CYP1A—one of the main PAHs’ biotransformation enzymes—induction, measured as increased ethoxyresorufin-O-deethylase (EROD) activity or increased mRNA levels [16,17], the disturbance of lipid metabolism [13] and oxidative stress [18].

Benzo(k)fluoranthene (BkF) is a high-molecular-weight 5-ring PAH, one of the 16 PAHs that the Environmental Protection Agency designated as priority contaminants, also belonging to the list of 8 PAHs that are categorized as a Group 2B carcinogen [19]. It has been found in freshwaters in concentrations of 7.0 ± 1.0 ng/L and 4.4 ± 1.1 ng/L in the Iberian Peninsula [20] and concentrations of 7.2 to 17.2 ng/L in Hungary [21]. Because of its high hydrophobicity, BkF strongly binds to organic materials in sediments of aquatic settings, becoming more resistant to degradation [22]. Moreover, due to its high lipophilicity, it can gradually accumulate and bioconcentrate in fish tissues [16,23].

The combined effects of temperature and PAHs have received little attention in ecotoxicology, and the literature mainly focuses on PAHs in complex mixtures. For example, in juvenile rainbow trout (*Oncorhynchus mykiss*) exposed, at 12 °C and 24 °C, to Rhine River sediment suspensions either enriched or not with a mixture of PAHs, mortality and PAH metabolization increased at 24 °C [24]. Hepatic EROD activity also increased in the liver of juvenile Atlantic cod (*Gadus morhua*) exposed to crude oil (72 h) at 2, 7 and 10 °C, with more pronounced induction at higher temperatures [25]. The effects of crude oil and temperature (4 °C vs. 11 °C) were also assessed in the liver of polar cod (*Boreogadus saida*) in a transcriptome study [26]. The gene expression of *CYP1A* was significantly upregulated, and this response was more potent at 11 °C. Curiously, the mRNA expression of the aryl hydrocarbon receptor repressor (*AhRr*) and heat shock proteins was higher at 4 °C.

The liver is a complex organ with high metabolic activity and involved in detoxifying countless substances [27,28]. As the primary site of PAH metabolism, the liver is a target organ for investigating their effects and the enzymatic pathways involved in their degradation [28,29]. Currently, in vitro testing is widely used in mechanistic and ecotoxicological studies. It is acknowledged that 2D models may not capture the complexities of multicellularity, and 3D systems such as spheroids better mimic in vivo conditions by promoting more natural cell-to-cell interactions and the creation of an extracellular matrix and by recreating nutrient and oxygen gradients [30,31]. Three-dimensional fish models have gained notoriety in the last decade, with spheroids being developed from trout primary hepatocytes [32,33,34] and liver-derived cell lines such as the rainbow trout RTL-W1 [35,36] and zebrafish ZFL [37,38]. Recently, a 3D model of the rainbow trout-liver-derived cell line RTL-W1 was implemented using ultra-low attachment (ULA) plates [35]. The model expressed higher *CYP1A* levels than the same cells cultured in a monolayer, and the *CYP1A* induction was higher in 3D than in 2D after exposure to the PAH benzo(α)pyrene (BaP). However, it should be noted that this study did not account for temperature variations.

The present study assessed the impacts of a model PAH, BkF, by exposing RTL-W1 spheroids, generated using ULA plates, to two concentrations of BkF (10 nM and 100 nM) over 4 days. Furthermore, the possible interaction between temperature and BkF was investigated by culturing spheroids at 18 °C and 23 °C. The isolated and joint effects of the two stressors were examined through the gene expression analysis of detoxification-related genes, as well as the viability of the spheroids, biometric parameters, and cell morphology. Our findings unveil independent and interactive effects, further sustaining that RTL-W1 spheroids are a powerful tool for ecotoxicological evaluations.

## 2. Materials and Methods

### 2.1. RTL-W1 Cell Culture and Spheroid Formation

RTL-W1 cells were cultured in T-75 cm^2^ culture flasks (5520200, Orange Scientific, Braine-l’Alleud, Belgium) at 18 °C in Leibovitz’s L-15 culture medium (21083-027, Gibco^TM^, Thermo Fisher Scientific, Grand Island, NY, USA) supplemented with 5% fetal bovine serum (FBS) (F9665, Sigma Aldrich, Saint Louis, MI, USA) and 1% penicillin/streptomycin (10,000 U/10,000 μg/mL) (A2213, Merck, Darmstadt, Germany). Cells with passages between 111 and 121 were used. The medium was renewed every 2 to 3 days for regular maintenance until cells reached confluency. For spheroid formation, confluent flasks were washed twice with phosphate-buffered saline (PBS) and trypsinized using a 0.05% trypsin, 0.02% ethylenediaminetetraacetic acid (EDTA) solution (59418C, Sigma-Aldrich, Saint Louis, MI, USA). Thereafter, cells were counted using a Neubauer chamber (0640031, Marienfeld, Lauda-Königshofen, Germany) to assess cell density after staining with trypan blue dye (0.4% in PBS). The cells were seeded in ULA 96-well round-bottom plates (4520, Corning, New York, NY, USA) at a density of 60,000 cells/well. The spheroids were cultured at 18 °C (Heraeus, BK 6160, Thermo Scientific, Waltham, MA, USA), and the medium (200 µL/well) was replaced every 2 to 3 days from the 3rd to the 10th day in culture.

### 2.2. Exposure Assays

Ten-day-old spheroids were exposed to BkF (CAS 207-08-9, Sigma-Aldrich, Saint Louis, MI, USA) at 18 °C or 23 °C. This timeframe was chosen based on the period, 7–21 days post-seeding, for functional studies in RTL-W1 spheroids [35], as well as for spheroids maintaining expression patterns of xenobiotic metabolism genes similar to whole livers after 10 days [34].

Five independent assays were conducted per temperature. For each temperature, the independent assays included the following exposure conditions: a control (C) made of the supplemented L-15 medium, a solvent control (SC) consisting of the supplemented L-15 medium with 0.1% dimethyl sulfoxide (DMSO), and two concentrations of BkF, 10 and 100 nM (BkF10 and BkF100), where the DMSO concentration was also kept at 0.1%. The exposure media were prepared independently for each experiment from a stock of BkF at 0.001 M in DMSO. All independent assays consisted of two plates, each including all BkF exposure conditions, comprising 24 spheroids per condition per plate (giving a total of 48 spheroids per exposure condition). In the 23 °C exposures, the spheroids were changed from the 18 °C (Heraeus, BK 6160, Thermo Scientific, Langenselbold, Germany) to the 23 °C incubator (INCU-Line 150R premium, VWR, Leuven, Belgium) immediately after exposure to BkF. The temperature range was chosen based on the optimal temperature for rainbow trout [39] and for culturing RTL-W1 cells (18 °C) [40]; since a 3 °C temperature increase had minimal impact in 2D-cultured RTL-W1 cells [40] and, moreover, increases in freshwater temperatures of the Iberian Peninsula are predicted to surpass 3 °C during heatwaves [41,42], a 5 °C increase (23 °C) was selected for the upper temperature in this study. The exposure period in culture was from the 10th to the 14th day, with media renewal on the 12th day. The 4-day exposure time, in addition to being within the period established for functional studies in RTL-W1 spheroids, aligns well with other toxicological studies using 2D-cultured RTL-W1 cells as a model for assessing cell viability and the expression of detoxification target genes [40,43].

### 2.3. Viability Assays

#### 2.3.1. alamarBlue

The alamarBlue™ HS Cell Viability Reagent (A50101, Thermo Fisher Scientific, Vantaa, Finland) was used. Harvested spheroids (*n* = 3 per condition/plate) were transferred by pipette aspiration to a 96-well plate (351172, Falcon, Corning, New York, NY, USA). Then, 90 µL of media from the respective exposure condition and 10 µL of the alamarBlue™ reagent were added to each of the wells. Background controls (*n* = 6 per condition) were carried out with media from the respective conditions without spheroids. Fluorescence was measured after 4 h of incubation with the alamarBlue™ reagent (18 °C or 23 °C), using an excitation wavelength of 550 nm and an emission wavelength of 588 nm in a Biotek Synergy™ HTX multimode microplate reader (Agilent, Santa Clara, CA, USA) using Gen5 software version 3.05.11 (Agilent, Santa Clara, CA, USA). The average relative fluorescence units (RFU) of the background controls (per respective condition) were calculated and subtracted from the individual samples. The sample values were then averaged per condition for the statistical analysis.

#### 2.3.2. Lactate Dehydrogenase (LDH) Assay

The LDH Cytotoxicity WST Assay kit (ENZ-KIT157-2500, Enzo Life Sciences, New York, NY, USA) was used to determine the LDH leakage in the cell culture supernatant. For each experiment (18 °C or 23 °C), 100 µL of media from spheroid housing wells (*n* = 3 per condition/plate) was collected. For the background controls, fresh media from respective exposure conditions were used (*n* = 6 wells per condition). The media were transferred to a 96-well plate (351172, Falcon, Corning, New York, NY, USA), and 100 µL of an assay buffer was added to each well. After the incubation (30 min, protected from light), 50 µL of a stop solution was added, and the absorbance was read at 490 nm in a MultiskanTM GO microplate spectrophotometer (Thermo Scientific, Waltham, MA, USA). For this assay, an average of the absorbances of the background controls (from each condition) was calculated and subtracted from the values of the individual samples. The sample values were then averaged per condition for the statistical analysis.

### 2.4. Biometric Parameters of Spheroids

To analyze the spheroids’ biometry after the exposures, images were taken with a stereomicroscope (SZX10, Olympus, Tokyo, Japan) coupled to a digital camera (DP21, Olympus, Tokyo, Japan). Biometric parameters (area, equivalent diameter and sphericity) were automatically analyzed at both temperatures (*n* = 24 spheroids per condition/plate) using the dedicated open-source software AnaSP (v. 2.0) [44]. All spheroids were examined, and the values were averaged per condition (*n* = 5 per temperature) for statistical analysis.

### 2.5. Optical Microscopy

In each experiment, the harvested spheroids were fixed in 10% buffered formalin (*n* = 3 spheroids per condition/plate) for 48 h and kept in 70% ethanol for at least 24 h. Then, the spheroids were embedded in Histogel (HG-4000-012, Epredia, Breda, The Netherlands) to be subjected to histological routine processing using an automatic tissue processor (TP1020, Leica Biosystems, Wetzlar, Germany). After processing, the spheroids were embedded in paraffin (6774006, Epredia, Breda, The Netherlands) in an embedding station (EG1140C, Leica Biosystems, Wetzlar, Germany). Using an automatic rotary microtome (RM2255, Leica Biosystems, Wetzlar, Germany), 3 μm sections were obtained. The spheroids were sectioned in their totality, and the following procedures were performed at different sectioning levels. The sections were routinely stained with hematoxylin and eosin, visualized in a microscope (BX50, Olympus, Tokyo, Japan) and photographed with a digital camera (EP50, Olympus, Tokyo, Japan).

### 2.6. Processing for Transmission Electron Microscopy [3]

Harvested spheroids (*n* = 3 per condition/plate) were fixed for 2 h (4 °C) in a solution of 2.5% glutaraldehyde (1.04239, Merck, Darmstadt, Germany) in a 0.1 M sodium cacodylate buffer (8.20670, Merck, Darmstadt, Germany), pH 7.2. After fixation, a TEM processing protocol was followed [45]. Briefly, the fixative was removed by two washing steps of 10 min with a 0.1 M cacodylate (8.20670, Merck, Darmstadt, Germany) buffer. Then, the spheroids went through a 2 h post-fixation with a solution of 1% osmium tetroxide (AGR1024, Agar Scientific, Essex, UK) in a 0.1 M sodium cacodylate buffer, pH 7.2, at 4 °C. After the post-fixation, spheroids were washed twice (10 min) in a 0.1 M cacodylate buffer, and a dehydration process was followed. The dehydration process included 30-min passages with increasing ethanol concentrations followed by two 15-min baths of propylene oxide (8.07027.1000, Merck, Darmstadt, Germany), and then they were placed in rising concentrations of epoxy resin diluted in propylene oxide until a final step of 100% resin. The spheroids were embedded in 100% epoxy resin in rubber embedding molds that were kept at 60 °C for 48 h to allow polymerization. The resin blocks were then manually trimmed with razor blades, and semithin sections were obtained in an ultramicrotome (EMUC7, Leica Biosystems, Hernalser Hauptstrasse, Germany) using diamond knives (Diatome, Nidau, Switzerland). The semithin sections (1 µm) were collected and stained (5 min) with a 1:1 mixture of 1% methylene blue (1.15943, Merck, Darmstadt, Germany) and 1% azure II (1.09211, Merck, Darmstadt, Germany) at 60 °C and, after washing with distilled water, dried at 60 °C and mounted with Coverquick 2000 (05547530, VWR Chemicals, Fontenay-sous-bois, France). The slides were observed and photographed using a microscope (BX50, Olympus, Tokyo, Japan) and a digital camera (EP50, Olympus, Tokyo, Japan). Ultrathin sections (90 nm) were generated using an ultramicrotome (EMUC7, Leica Biosystems, Vienna, Austria) with diamond knives (Diatome, Nidau, Switzerland) and adhered to 200 mesh copper grids. The grids were observed with a transmission electron microscope (100CXII, JEOL, Tokyo, Japan), and images were captured using a digital camera (Orius SC1000 CCD, Gatan, Pleasanton, CA, USA).

### 2.7. Semi-Quantitative Ultrastructure Analysis

A semi-quantitative analysis was conducted to more objectively perceive the relative abundance of the endoplasmic reticulum (smooth and rough), Golgi apparatus, lipid droplets, mitochondria, dense bodies, membrane protrusions, intermediate filaments and vacuolization. The semi-quantification followed a visual grading scale from 0 to 3, where 0 corresponded to “non-observed”, 1 to “present”, 2 to “moderately present”, and 3 to “very frequent” (*n* = 2 spheroids per temperature/condition). No differences in organelle content were noted between temperatures, so data from both temperatures were grouped by BkF exposure condition for the statistical analysis. To gain statistical power, data were further grouped as control (C + CS) versus treated (BkF10 + BkF100), making *n* = 8 spheroids per group.

### 2.8. Immunocytochemistry (ICC)

Slides (*n* = 3 spheroids per condition and temperature) were selected for staining with a polyclonal anti-CYP1A (CP-226) antibody (C02401201, Biosense Laboratories AS, Bergen, Norway). For that purpose, sections were first deparaffinized and hydrated through a series of decreasing ethanol concentrations and a 5-min running water wash step. Antigen retrieval was performed before incubation with the primary antibody. For that, a citrate buffer, pH 6.0, was pre-warmed in a pressure cooker; slides were then immersed and kept in a boiling citrate buffer at maximal pressure for 3 min. After, the slides were allowed to cool to room temperature and washed with distilled water. To block the endogenous peroxidase, slides were immersed for 10 min in 3% hydrogen peroxide (1.072101000, Merck, Darmstadt, Germany) in methanol (32213, Honeywell, Charlotte, NC, USA). From this step on, the Novolink^TM^ Max Polymer Detection System kit (Leica Biosystems, Wetzlar, Germany) was used following the manufacturer’s instructions. The primary antibody was diluted (1:500) in PBS with 5% bovine serum albumin (BSA, NZYTech, Lisbon, Portugal). A 3,3′-diaminobenzidine (DAB) solution was used as a chromogen, and the slides were counterstained with Mayer’s hematoxylin. The slides were dehydrated, mounted and observed in a light microscope (BX50, Olympus, Tokyo, Japan) equipped with a digital camera (EC50, Olympus, Tokyo, Japan).

### 2.9. RNA Extraction

A pool of spheroids from each condition (30 to 32 spheroids) was obtained in each experiment (18 °C and 23 °C) and centrifuged (Mega Star 600R, VWR, Leuvan, Belgium) at 200× *g* for 5 min to obtain a pellet that was frozen in liquid nitrogen at −80 °C up to RNA extraction using the illustra^TM^ RNAspin Mini kit (25-0500-72, GE Healthcare, Little Chalfont, UK). The concentration of the eluted RNA samples was determined by loading 2 µL of each sample into a µDrop Plate of a spectrophotometer (Multiskan Go, Thermo Scientific, Vantaa, Finland).

### 2.10. cDNA Synthesis and RT-qPCR

cDNA was synthesized with the iScriptTM Reverse Transcription Supermix for RT-qPCR (1708841, Bio-Rad, Hercules, CA, USA) in a T-Gradient Thermoblock (3112135, Biometra, GmbH, Gottingen, Germany). Each reaction comprised 0.6 μg of RNA template for a 20 µL final volume. The RT-qPCRs were run in a CFX ConnectTM Optics Module (Bio-Rad, Hercules, CA, USA) using the iQ SYBR^®^ Green Supermix (1708886, Bio-Rad, Hercules, CA, USA) and *CYP1A1*, *CYP3A27*, *GST*, *UGT*, *MRP2*, *BSEP*, *CAT* and *AhR*-specific primers. Elongation factor 1 alpha (*EF1α*) and beta-actin (*β-actin*) were used as reference genes to normalize the mRNA expression according to the Pfaffl method [46]. A melt curve analysis was also conducted to ensure product specificity. The primer sequences and qPCR conditions are presented in Table 1.

### 2.11. Statistical Analysis

The analysis was performed using the open-source software Jamovi (v. 2.3). The Shapiro–Wilk and Levene tests were used to assess normality and homogeneity of variances assumptions, respectively. If needed, data were transformed to meet the assumptions to perform some parametric tests. For the statistical analysis of the spheroids’ biometry, viability (LDH and alamarBlue assays) and gene expression, data were analyzed with a two-way ANOVA, using Tukey’s test as a post-hoc. For the semi-quantitative ultrastructure, statistical differences were analyzed using a non-parametric one-way ANOVA (Kruskal–Wallis), followed by Dwass–Steel–Critchlow–Fligner pairwise comparisons. The significance level was set at 0.05. Graphs were made in GraphPad Software (v. 7.04).

## 3. Results

### 3.1. Viability Assays

**Figure 1:**
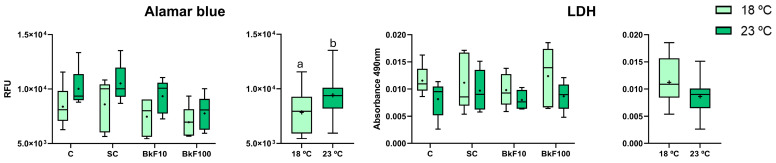
alamarBlue and lactate dehydrogenase (LDH) assay data for RTL-W1 spheroids after a 4-day exposure (from day 10 to day 14 in culture) to benzo(k)fluoranthene (BkF): C—control; SC—solvent control; BkF10—10 nM of BkF; BkF100—100 nM of BkF at 18 °C and 23 °C. Data in boxplots correspond to the median, mean (+), interquartile range, minimum and maximum, displayed by condition on the left panel and grouped by temperature on the right. Relative fluorescence units (RFU) and absorbance values (490 nm) are shown against each condition and temperature. Different letters (a and b) illustrate significant temperature differences according to a two-way ANOVA followed by Tukey’s test (*p* < 0.05). *n* = 5 independent assays per temperature.

The exposed spheroids were always integrous, independently of the BkF exposure condition (Supplementary Figure S1). In agreement, the alamarBlue and LDH assays did not show significant differences between exposure conditions (Figure 1). Overall, the spheroids remained viable, even at the highest concentration of BkF (100 nM). However, regarding alamarBlue, the effect of temperature alone was significant, and RFU levels were higher at 23 °C than at 18 °C. LDH values were not significantly altered by the temperature increase.

### 3.2. Biometric Parameters

**Figure 2:**
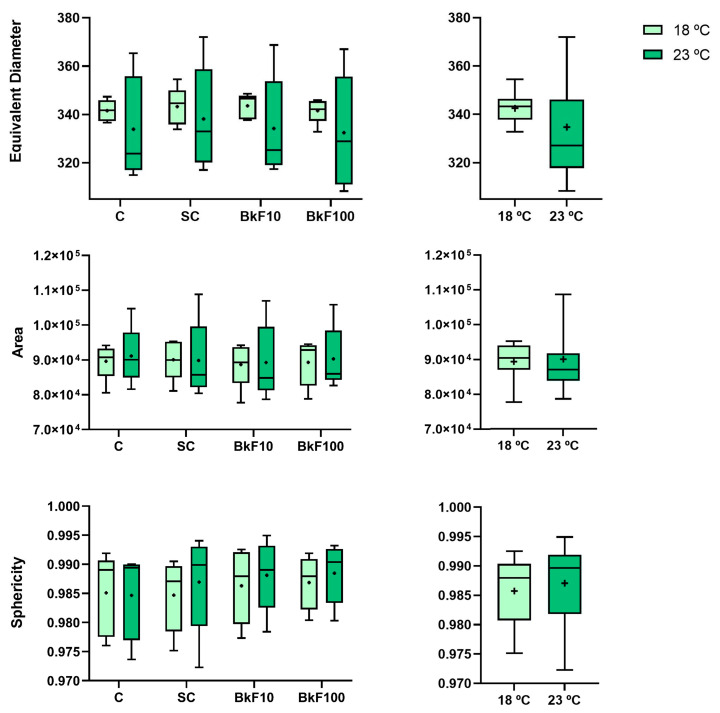
Equivalent diameter (µm), area (µm^2^) and sphericity of RTL-W1 spheroids after a 4-day exposure (from day 10 to day 14 in culture) to benzo(k)fluoranthene (BkF): C—control; SC—solvent control; BkF10—10 nM of BkF; BkF100—100 nM of BkF at 18 °C and 23 °C. Data correspond to the median, mean (+), interquartile range, minimum and maximum, displayed by condition on the left panel and grouped by temperature on the right. *n* = 5 independent assays per temperature.

The analyses of the biometry results (Figure 2) showed that neither the exposure to BkF nor increasing temperatures significantly influenced the spheroids’ area, equivalent diameter or sphericity.

### 3.3. Optical Microscopy of Paraffin Sections

**Figure 3:**
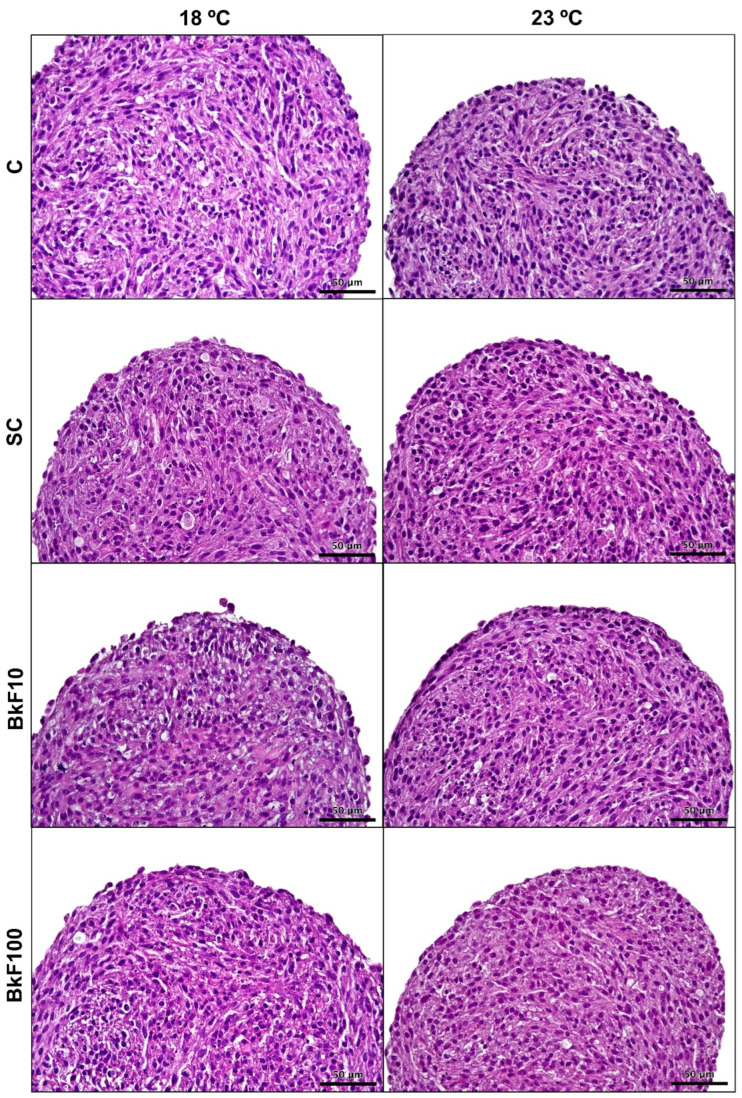
Hematoxylin and eosin-stained histological sections of RTL-W1 spheroids after a 4-day exposure (from day 10 to day 14 in culture) to benzo(k)fluoranthene (BkF) at 18 °C and 23 °C: C—control; SC—solvent control; BkF10—10 nM of BkF; BkF100—100 nM of BkF.

The spheroids (Figure 3) were formed of roundish cells mixed with more elongated ones, frequently forming nest arrangements in their core. Cytoplasmic vacuoles and occasional apoptotic figures were also observed. The spheroids’ border was always well-defined of either roundish or elongated cells. Necrotic cores were never observed. The described spheroids’ overall structure remained unchanged after BkF exposure (Figure 3 and Supplementary Figure S2), and no noticeable modifications were observed between 18 °C and 23 °C.

### 3.4. Optical Microscopy of Semithin Sections and Transmission Electron Microscopy

**Figure 4:**
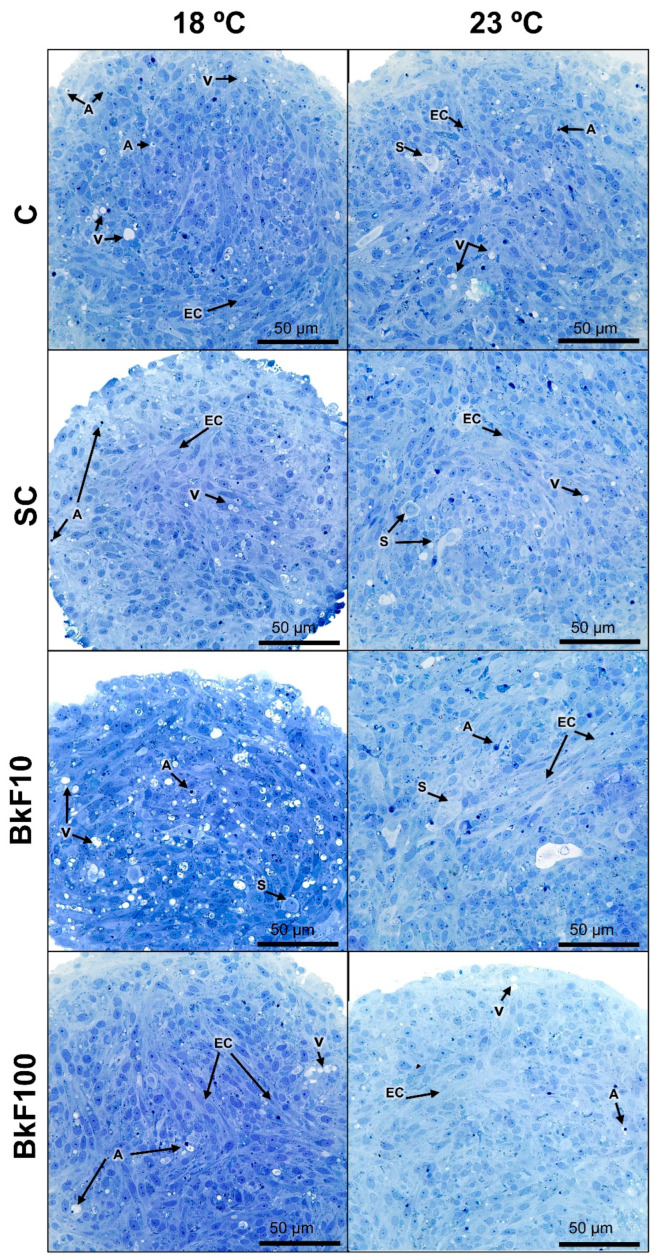
Semithin sections of RTL-W1 spheroids after a 4-day exposure (from day 10 to day 14 in culture) to benzo(k)fluoranthene (BkF) at 18 °C and 23 °C: C—control; SC—solvent control; BkF10—10 nM of BkF; BkF100—100 nM of BkF. A—pyknotic nuclei; EC—elongated cells; S—senescent cells; V—vacuoles.

The observation of semithin sections before TEM analysis revealed no discernible variations in the spheroids’ morphology across conditions and temperatures (Figure 4 and Supplementary Figure S3). A steady population of cells with more translucent and less stained cytoplasm (putative senescent cells) could also be identified (Figure 4). Moreover, there were scattered cells with intact profiles but with pyknosis.

**Figure 5:**
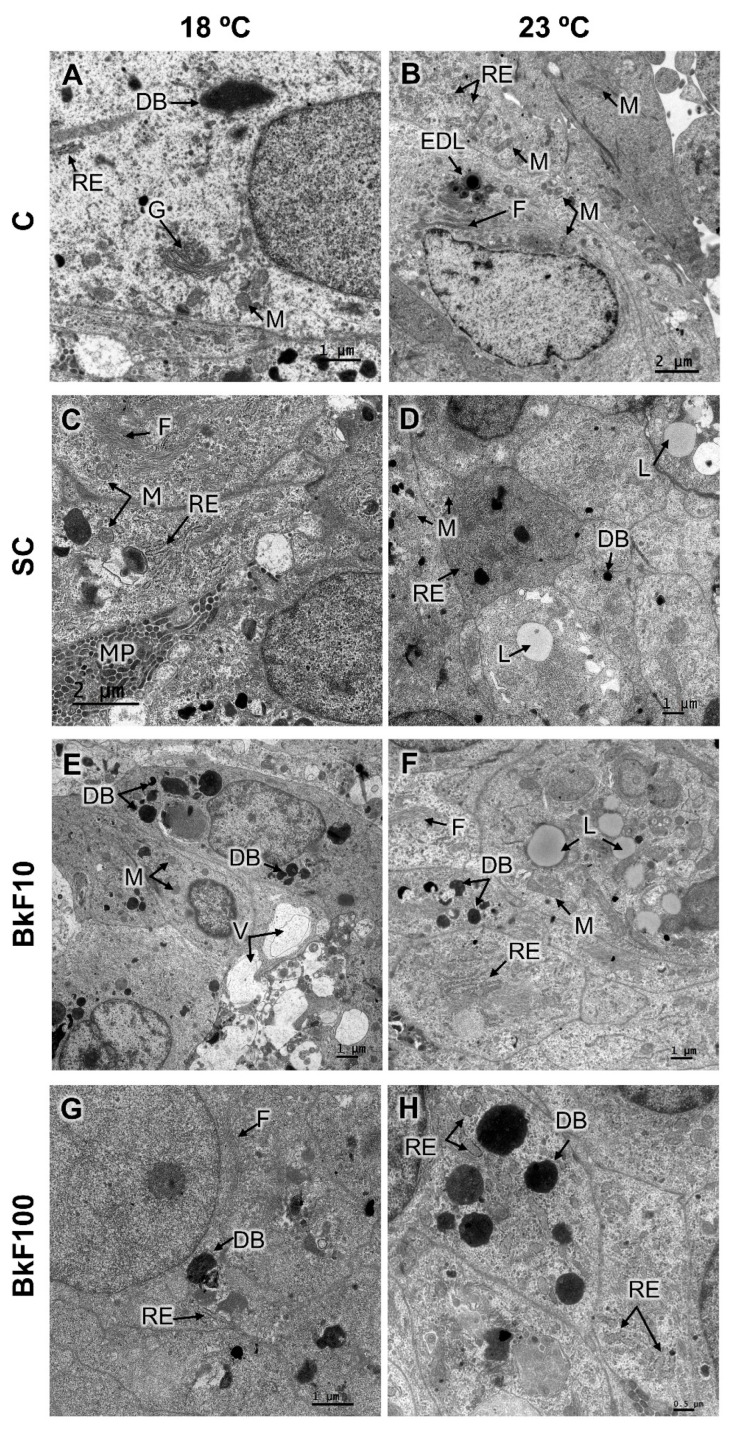
Ultrathin sections of RTL-W1 spheroids after a 4-day exposure (from day 10 to day 14 in culture) to benzo(k)fluoranthene (BkF) at 18 °C and 23 °C: C—control; SC—solvent control; BkF10—10 nM of BkF; BkF100—100 nM of BkF. (**A**)—C (18 °C); (**B**)—C (23 °C); (**C**)—SC (18 °C); (**D**)—SC (23 °C); (**E**)—10 nM BkF (18 °C); (**F**)—10 nM BkF (23 °C); (**G**)—100 nM BkF (18 °C); (**H**)—100 nM BkF (23 °C). DB—dense bodies; EDL—electron-dense lipids; F—intermediate filaments; G—Golgi apparatus; L—electron-lucent lipids; M—mitochondria; MP—membrane protrusions; RE—endoplasmic reticulum.

At the TEM level (Figure 5), the endoplasmic reticulum (typically rough) and mitochondria were the most abundant organelles. Dense bodies were relatively abundant in all conditions, including controls (Figure 5A), but at 23 °C and BkF-treated conditions, they appeared to form larger agglomerates (Figure 5H). The Golgi apparatus, albeit less common, could occasionally be identified (Figure 5A). Elongated membrane protrusions were frequently observed in all settings but were more noticeable at 18 °C under control conditions (Figure 5C). Lipid droplets were observed in all cases in two forms: one more electron-dense (Figure 5B) and the other more electron-lucent (Figure 5D). Intermediate filaments (Figure 5B,C,F,G) were common in all situations. Vacuoles were consistently present but more commonly in the treated conditions (Figure 5E). At 23 °C, endoplasmic reticulum cisterns were dilated more frequently (Figure 5H, Supplementary Figure S4).

### 3.5. Semi-Quantitative Ultrastructural Analysis

**Figure 6:**
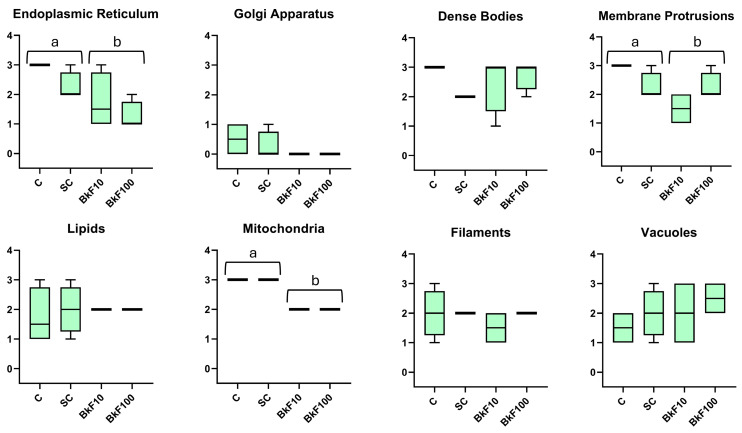
Grading of the frequency of selected ultrastructural aspects of RTL-W1 spheroids after a 4-day exposure to benzo(k)fluoranthene (BkF): C—control; SC—solvent control; BkF10—10 nM of BkF; BkF100—100 nM of BkF. Values range from 0 (undetected) to 3 (very frequent). Data are presented in boxplots as median, interquartile range, minimum and maximum. Different letters (a vs. b) illustrate significant differences between control and treated conditions (joined under horizontal brackets) according to a one-way ANOVA (Kruskal–Wallis) followed by Dwass–Steel–Critchlow–Fligner pairwise comparisons (*p* < 0.05).

A summary of the semi-quantitative results can be seen in Supplementary Table S1. The frequency of endoplasmic reticulum, mitochondria and membrane protrusions diminished in the treated conditions (Figure 6). There was also a trend for increased frequency of vacuoles in the treated conditions.

### 3.6. CYP1A Immunocytochemistry

**Figure 7:**
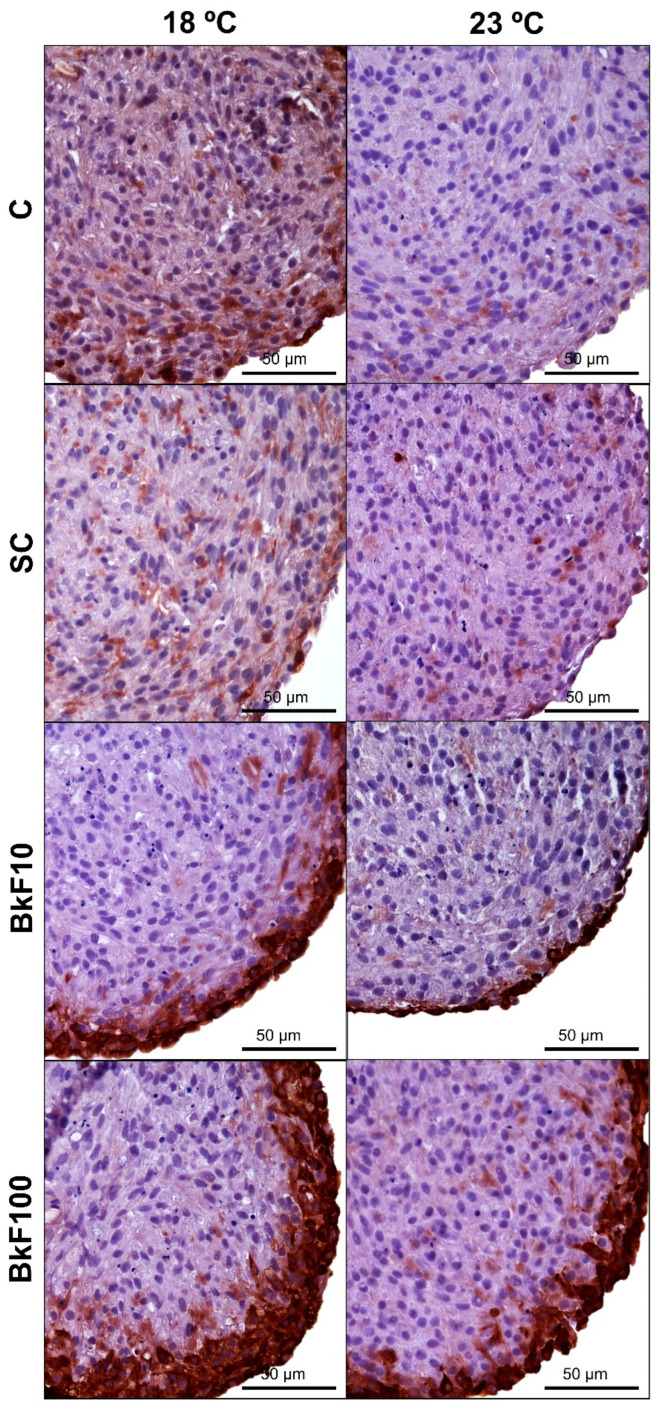
Immunocytochemistry labelling of RTL-W1 spheroids with anti-CYP1A antibody after a 4-day exposure (from day 10 to day 14 in culture) to benzo(k)fluoranthene (BkF) at 18 °C and 23 °C.

There was widespread weak positive immunostaining in the control groups, with sporadic and strongly tagged cells distributed throughout the spheroids (Figure 7 and Supplementary Figure S4). At both temperatures, exposure to BkF significantly enhanced the CYP1A signal, with the protein expression showing a dose-dependent increase, as spheroids exposed to 100 nM BkF exhibited the strongest labelling. In the BkF-exposed conditions, although staining was dispersed throughout the spheroids, it was primarily localized on the periphery (Figure 7).

### 3.7. Gene Expression

**Figure 8:**
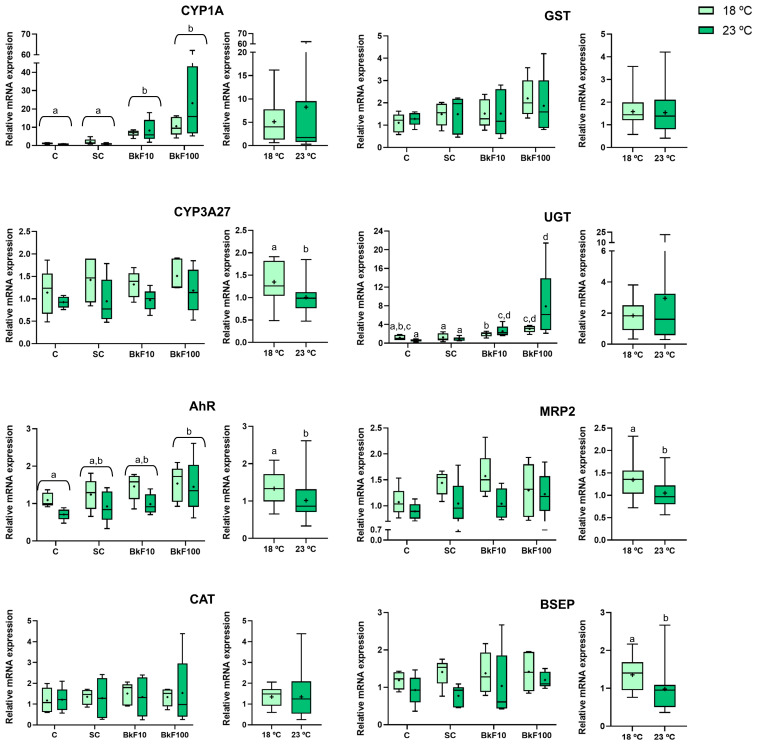
Relative gene expression of detoxification target genes (*CYP1A*, *CYP3A27*, *AhR*, *CAT*, *GST*, *UGT*, *MRP2*, and *BSEP*) in RTL-W1 spheroids after 4 days of exposure (between days 10 and 14 in culture) to benzo(k)fluoranthene (BkF) at 18 °C and 23 °C: C—control; SC—solvent control; BkF10—10 nM of BkF; BkF100—100 nM of BkF. Data are presented in boxplots as median, mean (+), interquartile range, minimum and maximum, displayed by condition on the left panel and grouped by temperature on the right. Conditions (joined under horizontal brackets) not sharing a common letter (a or b) differ significantly according to a two-way ANOVA followed by Tukey’s test (*p* < 0.05). *AhR—*aryl hydrocarbon receptor; *BSEP*—bile salt export pump; *CAT*—catalase; *CYP1A*—cytochrome P450 1A; *CYP3A27*—cytochrome P450 3A27; *GST*—glutathione S-transferase; *MRP2*—multidrug resistance-associated protein; *UGT*—uridinediphosphate (UDP)–glucuronosyltransferase.

Both phase I and II of the detoxification genes were impacted by exposure to BkF and/or increasing temperatures (Figure 8). *CYP1A* mRNA expression was upregulated in both BkF concentrations (10 and 100 nM), and the increase seemed dose-dependent, especially at 23 °C. Despite this, no interaction effects between temperature and BkF exposure were noted. An interaction effect was, however, obtained in *UGT* mRNA expression. There was a significant increase in this gene expression at the higher BkF concentration (100 nM) compared with both controls, but only at 23 °C. The *AhR* gene expression was significantly upregulated at the highest PAH concentration (100 nM) compared with the control, and it was downregulated at 23 °C. Both xenobiotic transporters (*MRP2* and *BSEP)* and the phase I *CYP3A27* gene significantly decreased mRNA levels at 23 °C, with no effect observed at either PAH concentration. The *CAT* and *GST* genes showed stable mRNA levels in all exposure conditions.

## 4. Discussion

This work used RTL-W1 spheroids to study the effects of BkF, a model PAH, and temperature in the fish liver detoxification pathways, advancing the development of 3D in vitro models to analyze the impact of aquatic pollutants in a warming world. The spheroids reacted to BkF and temperature stress with changes in the expression of detoxifying genes and ultrastructure.

The spheroids maintained their integrity despite exposure to BkF and temperature stress. In agreement, the LDH levels remained stable after both stressors, indicating that the membranes’ integrity was not compromised. However, there was a slight but not significant decreasing trend in RFU levels after BKF exposure, suggesting that BkF was on the verge of impacting or starting to alter the spheroids’ metabolization capacity. At 23 °C, however, the significantly greater RFU values (compared to 18 °C) indicated increased metabolization at the highest temperature. In line with our data, warmer conditions in fish were linked to an increased biotransformation capacity in vivo. In polar cod (*Boreogadus saida*) exposed to naphthenic crude oil (starting concentration of 67 mg oil/L) at 4 and 11 °C, the oil weathered more quickly, and the concentration of 2- and 3-ring PAHs decreased more rapidly at the higher temperature [26]. Similarly, the metabolite profiles of bluegill sunfish (*Lepomis macrochirus*) acclimated to 13 and 23 °C revealed that BaP biotransformation occurred at slower rates at the lower temperature [51]. Here, the spheroids’ biometrics also reached stable values after the exposures. Independently of the temperature or exposure conditions, all spheroids appeared compact, without signs of disaggregation.

In the optical microscopic analysis of paraffin and epoxy resin sections, the morphology of the spheroids showed intact nuclei and the absence of evident signs of cellular damage in addition to the nest-like arrangements typical for this cell line [52]. No significant levels of cell structural damage could be distinguished in either stressor. The TEM analysis showed that the spheroids’ cells resembled, to some extent, differentiated trout hepatocytes [53,54]. The organelle aspects matched those reported in other studies using 3D-cultured RTL-W1 cells [35,36]. However, it was found here that exposure to BkF diminished the frequency of endoplasmic reticulum, mitochondria and membrane protrusions. It is worth noting that the Golgi complex was never seen in the treated conditions, which should not be only related to its scarcity in RTL-W1 cells. It has been known for a long time that the Golgi complex may organize and reorganize and that at least drugs or mutations may cause the loss of recognizable Golgi structures [55,56]. Moreover, Golgi complex disappearance occurs during apoptosis [57], and it was proposed that Golgi disassembly sets off a signaling pathway related to stress [58]. In line with our findings with BkF, it was acknowledged that BaP also strongly impacts both the distribution and functionality of the cell organelles. For example, in common carp (*Cyprinus carpio*) exposed to a concentration range of 100–600 μg/L BaP, an increase in lipid droplets and lysosomes was evidenced in hepatocytes [59]. In the latter study, the altered hepatocytes presented distorted mitochondria, damaged endoplasmic reticulum, and disrupted cell membranes, and such effects were dose-dependent [59]. Here, if, on the one hand, BkF exposure affected the organelle content of RTL-W1 spheroids, the temperature also affected their ultrastructure. At 23 °C, large aggregates of dense bodies and dilated RER cisterns were seen, which highly indicates hepatocyte injury [60,61]. Under in vivo heat stress (24 °C), rainbow trout showed morphological liver changes, including vacuolation, steatosis, reduced and disorganized mitochondrial cristae and slightly dilated endoplasmic reticula [62]; these alterations were linked to accumulated oxidative damage.

This study targeted gene expression across all xenobiotic detoxification stages and PAH-activated genes. Both BkF concentrations increased *CYP1A* mRNA levels, especially at the higher concentration (23 °C). This result aligns with the literature that reported CYP1A induction after PAH exposure. For example, 1 µM of BkF raised *CYP1A* mRNA levels and induced EROD in 2D-cultured RTL-W1 cells [17]. Also, in 2D cultures of RTL-W1, BkF was deemed a potent EROD inducer with an EC50 value of 8.28 nM [63]. It has also been demonstrated in primary hepatocytes of gilthead sea bream (*Sparus aurata*) that CYP1A is sensitive to PAH exposure, as evidenced by the elevation of the CYP1A1 gene and protein expression following a 24 h exposure to BaP at 0.1, 10 and 50 μM [64].

Three-dimensional cultures of liver cell lines have been shown to express higher levels of CYP1A than monolayers [35]. Therefore, they should be expected to obtain stronger inductions in 3D than in their 2D counterparts. This has been verified in RTL-W1 spheroids exposed to the PAH-like compound beta-naphthoflavone (β-NF). In contrast to cell monolayer cultures, spheroids exposed to β-NF (200 nM, 24 h) had maximum EROD induction levels with patterns linked to *CYP1A* expression [35]; yet, the 3D versus 2D differences were not so striking after exposure to BaP (1 μM for 24 h). Also, at the protein level, CYP1A was responsive to PAH exposure, with increased levels in a 3D culture of the fish hepatoma cell line PLHC-1 exposed to BaP (5 µM) [65]. The present study’s ICC on RTL-W1 spheroids corroborated the *CYP1A* mRNA expression levels, with the immunostaining signal increasing with the BkF concentration. The immunolabelling was weak and diffuse in controls, whereas, in BkF-treated spheroids, intense staining was observed at the periphery. Our results align with those of Rodd et al. [65], which showed increased CYP1A protein expression at the edge of microtissues exposed to 1 µM of BaP, with decreasing levels towards the center of the 3D aggregates. A likely explanation for this labelling pattern might be that the spheroids’ peripheral cells are more exposed to the tested toxicants. Herein, temperature alone did not affect *CYP1A* expression. Such a result was obtained in the RTL-W1 cell line cultured in 2D, where a 3 °C difference (18 and 21 °C) did not affect *CYP1A* mRNA expression [40]. The same pattern was found in vivo in zebrafish (*Danio rerio*), with no differences in the hepatic protein expression of CYP1A in fish exposed to 27 °C and 30 °C [66].

As per the other detoxification phase I enzyme, *CYP3A27*, the mRNA levels remained stable after exposure, while a downregulation was obtained at 23 °C. In contrast, CYP3A enzyme activity—evaluated by benzyloxy-4-trifluoromethylcoumarin-O-debenzyloxylase activity—increased in 2D cultures of RTL-W1 cells after 7 days exposure to BkF; effective concentrations ranging from 0.01 to 1 µM resulted in an EC50 of 0.3 µM [17]. In addition to the difference in target levels (gene expression vs. enzyme activity), the use of a 3D RTL-W1 model in the present study may have also contributed to the seeming discrepancy.

An interaction between temperature and BkF was seen in *UGT* mRNA expression levels. The BkF-exposed cells showed increased *UGT* expression, which was more evident in the highest concentration (100 nM) and at 23 °C. Previously, a 3 °C increase in 2D-cultured RTL-W1 cells could not alter (alone) the *UGT*’s expression [40]. Regarding the single PAH exposure, in a study with the Pacific white shrimp (*Litopenaeus vannamei*), UGT activity in the hepatopancreas was positively correlated with the BaP exposure concentration [67]. Furthermore, in Gulf killifish (*Fundulus grandis*) larvae, exposure to crude oil (0.15 μg/L) upregulated *UGT1A1* mRNA expression levels, although a high temperature alone resulted in the downregulation of this gene [68]. However, larvae co-exposure to a high temperature (30 °C vs. 20 °C) and oil upregulated *UGT1A1* [68]. Overall, our data, along with previous findings, suggest that *UGT* mRNA expression and/or activity can be simultaneously influenced by BkF and temperature.

Although no differences were noted here in *GST*’s and *CAT*’s mRNA levels following exposure to both stressors, *GST* expression was altered by increasing temperature in 2D-cultured RTL-W1 cells, peaking at 21 °C compared with 18 °C [40]. In agreement with the present results with spheroids, liver GST activity, measured at 1–120 h, was unaffected by the acute transfer of goldfish (*Carassius auratus*) from 3 to 23 °C [69]. In the same work, however, CAT enzymatic activity in the liver declined over time, reaching 55% of its starting value after 48 h [69]. In contrast, in carp (*Cyprinus carpio*), the liver activity of GST increased after BaP exposure (50–600 μg/L); at the lowest BaP concentration, GST levels increased only after 10 days of exposure, whereas at higher concentrations, the increase was observed as early as 2 days [59]. Moreover, in polar cod exposed to BaP (by intraperitoneal injection), GST and CAT increased; however, they had variable induction patterns; while increased expression of *GST* mRNA was seen on day 4, the highest upregulation of *CAT* was noted on day 2 [70]. The protein levels and enzymatic activities of the same targets showed delayed and more erratic responses compared with the changes in mRNA levels. From the above, it can be inferred that these antioxidant biomarker responses may vary between experimental models, depending on the measurement method (mRNA, protein, enzymatic activity) and the assessed time points.

The *AhR* gene was the only gene whose expression in RTL-W1 cells was altered by exposure to PAH and temperature. While the expression augmented with the highest BkF concentration, mRNA levels decreased at 23 °C. Earlier, RTL-W1 cells exposed in monolayers to 1 µM of BkF (24 h) also exhibited a significant increase in *AhR* mRNA expression [17]. The detoxification of AhR ligands is mediated via the AhR-triggered pathway. Therefore, AhR is a main regulator of xenobiotic detoxification, orchestrating the expression of several detoxification enzymes, namely CYP, GST and UGT. It is therefore noteworthy that its expression can be changed by temperature as happened here, a result that should be explored in future studies.

Here, at the higher temperature, the mRNA levels of both efflux transporters, *MRP2* and *BSEP*, were downregulated. This finding suggests that higher temperatures may deregulate efflux mechanisms, eventually reducing an organism’s ability to eliminate xenobiotics. In this cell line cultured in a monolayer, a 3 °C rise caused a non-significant trend towards increased *MRP2* mRNA levels at a higher temperature [40]. Despite the differences in 2D and 3D cultures, the overall results suggest that temperature affects xenobiotic clearance, warranting further investigation in future studies.

The findings here support the notion that 3D RTL-W1 spheroids may be explored as a model for researching the effects of heat stress, PAHs, and their interactions. It was unveiled that both BkF and temperature impacted gene expression in the covered detoxification phases. Furthermore, it was shown that PAHs and high temperatures can have interaction effects, as evidenced in the *UGT’*s mRNA expression levels. BkF affected the distribution of organelles and the mRNA expression of key detoxification genes (*CYP1A1* and *AhR*). Additionally, temperature affected the expression of key detoxification components (*CYP3A27*, *AhR*, *MRP2*, and *BSEP*) and influenced the ultrastructural characteristics of the spheroids. Such changes in the organelle content after PAH exposure should translate into impacts on cellular functioning [62]. Actually, the morphological alterations at the higher temperature (23 °C), such as the dilated endoplasmic reticulum cisterns, combined with the variations in gene expression, namely the decrease in the mRNA expression of *AhR*, *CYP3A27*, and efflux transporters, may help to understand the impacts of temperature stress at the cellular level.

## 5. Conclusions

The current work demonstrated the RTL-W1 spheroids’ applicability, sensitivity, and specific response to temperature stress, BkF, and PAH. At both studied concentrations (10 and 100 nM of BkF), effects were detected, particularly the stimulation of *CYP1A*, *AhR* and *UGT* gene expression, as well as increased CYP1A protein expression. The frequency of organelles was also affected by PAH exposure, with less frequent mitochondria, endoplasmic reticulum, and membrane protrusions on the BkF-treated spheroids. The higher temperature also increased the spheroids’ metabolization ability (greater RFU values) and downregulated *CYP3A27*, *AhR*, *MRP2*, and *BSEP* gene expression. The model employed here could be an alternative tool for identifying the combined effects of temperature and PAH exposure. For future research, it would be valuable to use the model to explore the effects of other PAHs or complex PAH mixtures, investigate additional pathways linked to PAH effects, such as oxidative stress and lipid metabolism, and examine lesser-known toxicity mechanisms of PAHs. Moreover, future studies could consider a range of exposure times and assess the dynamics of changes in gene expression at different time points.

## Figures and Tables

**Table 1:** Primer sequences used for the target genes with detailed protocol (annealing temperatures in bold) and amplification efficiencies.

Gene	Sequence	Protocol	Efficiency	References
*CYP1A*	F: GATGTCAGTGGCAGCTTTGA R: TCCTGGTCATCATGGCTGTA	95 °C—3 min (95 °C—20 s; **60.0 °C**—20 s; 72 °C—20 s) 40× 95 °C—1 min	97.7	[34]
*CYP3A27*	F: GACGGTGGAGATCAACG R: GAGGATCTCGACCATGG	95 °C—3 min (95 °C—20 s; **60.0 °C**—20 s; 72 °C—20 s) 40× 95 °C—1 min	99.0	[34]
*GST*	F: AGCTGCTCCCAGCTGATCC R:CAAACCACGGCCACATCATGTAATC	94 °C—3 min (94 °C—20 s; **60.0 °C**—20 s; 72 °C—20 s) 40× 94 °C—1 min	93.9	[47]
*CAT*	F: CACTGATGAGGGCAACTGGG R: CTTGAAGTGGAACTTGCAG	95 °C—3 min (95 °C—10 s; **58.0 °C**—30 s; 72 °C—30 s) 40× 95 °C—30 s	104.5	[48]
*UGT*	F: ATAAGGACCGTCCCATCGAG R: ATCCAGTTGAGGTCGTGAGC	94 °C—3 min (94 °C—20 s; **60.0 °C**—20 s; 72 °C—20 s) 40× 94 °C—1 min	94.9	[34]
*MRP2*	F: CCATTCTGTTCGCTGTCTCA R: CTCGTAGCAGGGTCTGGAAG	94 °C—3 min (94 °C—20 s; **60.0 °C**—20 s; 72 °C—20 s) 40× 94 °C—1 min	100.2	[34]
*BSEP*	F: CCGACCAGGGCAAAGTGATT R: CAGAATGGGCTCCTGGGATAC	94 °C—3 min (94 °C—20 s; **60.0 °C**—20 s; 72 °C—20 s) 40× 94 °C—1 min	95.3	[34]
*AhR*	F: GGATGCCACTGAGTTCCAAACCAA R: AATGCCTGGTCTATGGGTAGCTGA	95 °C—3 min (95 °C—20 s; **60.0 °C**—20 s; 72 °C—20 s) 40× 95 °C—1 min	101.6	[47]
*Ef1α*	F: TGCCACACTGCTCACATC R: TCTCCAGACTTCAGGAACTTG	94 °C—3 min (94 °C—20 s; **55.0 °C**—20 s; 72 °C—20 s) 40× 94 °C—1 min	97.4	[49]
*β-actin*	F: TCTGGCATCACACCTTCTAC R: TTCTCCCTGTTGGCTTTGG	94 °C—3 min (94 °C—20 s; **55.0 °C**—20 s; 72 °C—20 s) 40× 94 °C—1 min	99.7	[50]

*CYP1A*—cytochrome P450 1A; *CYP3A27*—cytochrome P450 3A27; *GST*—glutathione S-transferase; *CAT*—Catalase; *UGT*—uridinediphosphate (UDP)–glucuronosyltransferase; *MRP2*—multidrug resistance-associated protein 2; *BSEP*—bile salt export pump; *AhR*—aryl hydrocarbon receptor; *Ef1α*—elongation factor 1 alpha; *β-actin*—beta-actin.

## Data Availability

The original contributions presented in this study are included in the article/supplementary material. Further inquiries can be directed to the corresponding author.

## Supplementary Materials

The following supporting information can be downloaded at: https://www.mdpi.com/article/10.3390/toxics13040302/s1, Figure S1: Representative images of the spheroids on the plate wells. A—Spheroid from the control (C) condition on the 14th day, at 18 °C; B—Spheroid from control (C) condition on the 14th day, at 23 °C. C—Spheroid exposed to 100 nM of BkF (BkF100) on the 14th day, at 18 °C; D—Spheroid exposed to 100 nM of BkF (BkF100) on the 14th day, at 23 °C.; Figure S2: Hematoxylin and eosin-stained histological sections of RTL-W1 spheroids, after a 4-day exposure (from day 10 to day 14 in culture) to benzo(k)fluoranthene at 18 °C and 23 °C. C—Control; SC—Solvent Control; BkF10—10 nM of BkF; BkF100—100 nM of BkF; Figure S3: Semithin sections of RTL-W1 spheroids, after a 4-day exposure (from day 10 to day 14 in culture) at 18 °C and 23 °C to Benzo(k)fluoranthene (BkF). C—Control; SC—Solvent Control; BkF10—10 nM of BkF; BkF100—100 nM of BkF; Figure S4: Ultrastructural changes in RTL-W1 spheroids (A-D) after a 4-day exposure (from day 10 to day 14 in culture) to 10 nM of benzo(k)fluoranthene (BkF) at 23 °C. DB—Dense bodies accumulation; DER—Dilated endoplasmic reticulum; LB—Lamellar bodies deposition; M—Mitochondria; Figure S5: CYP1A immunocytochemistry labelling of RTL-W1 spheroids after a 4-day exposure (from day 10 to day 14 in culture) to Benzo(k)fluoranthene at 18 °C and 23 °C. C—Control; SC—Solvent Control; BkF10—10 nM of BkF; BkF100—100 nM of BkF; Table S1: Frequency of ultrastructural elements of RTL-W1 spheroids after a 4-day exposure (from day 10 to day 14 in culture) to benzo(k)fluoranthene (BkF). Grading scale: 0 (non-observed), 1 (present), 2 (moderately present), and 3 (very frequent). C—Control; SC—Solvent Control; BkF10—10 nM of BkF; BkF100—100 nM of BkF.
