# Supplementary material for: Effects of Benzo[k]fluoranthene at Two Temperatures on Viability, Structure, and Detoxification-Related Genes in Rainbow Trout RTL-W1 Cell Spheroids
Source: Toxics. 2025 Apr 12;13(4):302. doi: 10.3390/toxics13040302 (PMC12031258; doi:10.3390/toxics13040302)
Supplement: Supplementary file 1 [file toxics-13-00302-s001.zip › toxics-3555699-supplementary.pdf]

toxics

MDPI

Supplementary Materials

Effects of Benzo[k]fluoranthene at Two Temperatures on Viability, Structure, and Detoxification-Related Genes in Rainbow Trout RTL-W1 Cell Spheroids

Telma Esteves [1,2], Fernanda Malhão [1,2], Eduardo Rocha [1,2,*,†] and Célia Lopes [1,2,†]

[1] Laboratory of Histology and Embryology, Department of Microscopy, ICBAS - School of Medicine and Biomedical Sciences, University of Porto, Rua Jorge Viterbo Ferreira 228, 4050-313 Porto, Portugal
[2] Group of Animal Morphology and Toxicology, Interdisciplinary Centre of Marine and Environmental Research (CIIMAR/CIMAR) University of Porto, Terminal de Cruzeiros do Porto de Leixões, Av. General Norton de Matos s/n, 4450-208 Matosinhos, Portugal
† Joint last senior authors.
* Correspondence: to whom correspondence should be addressed

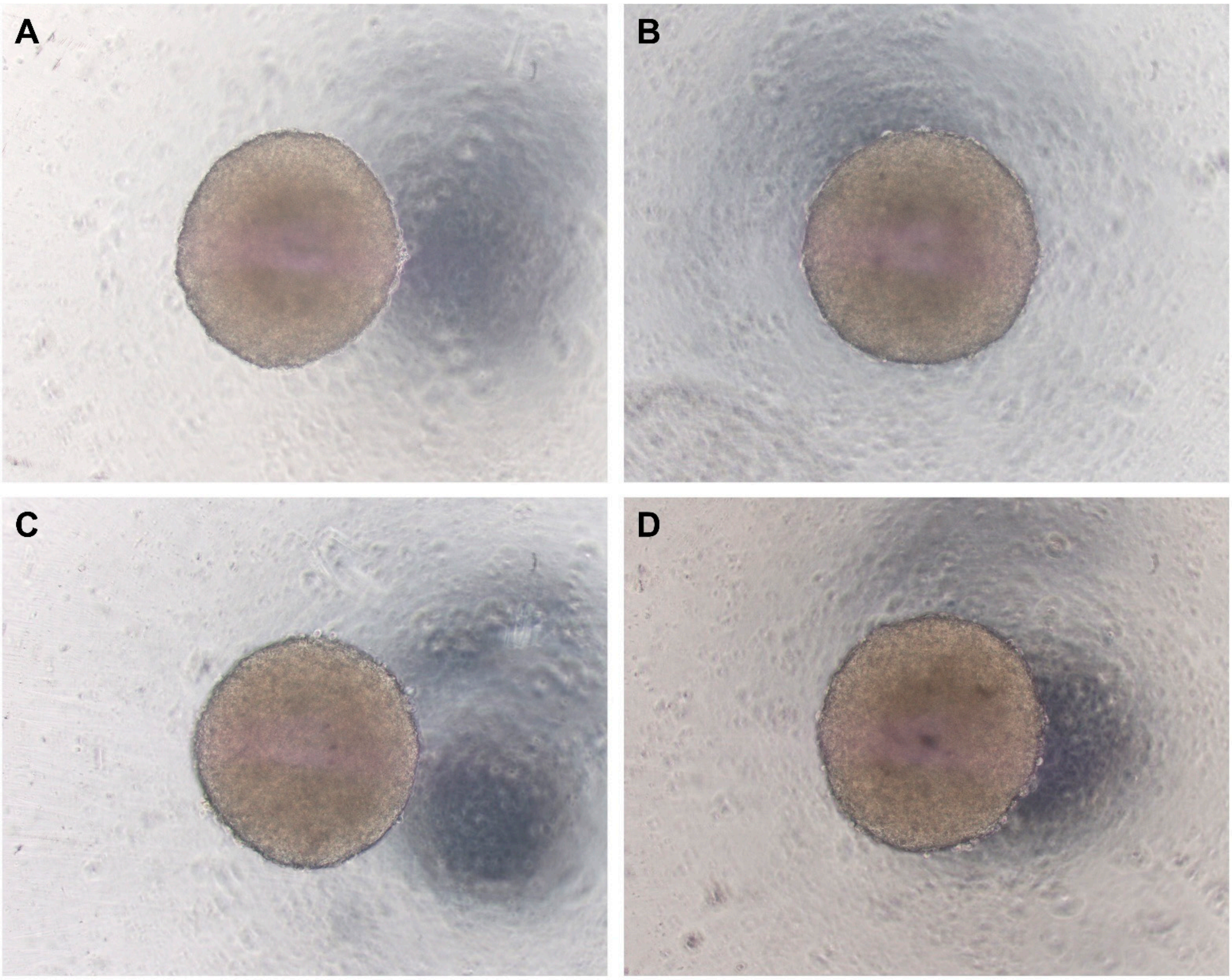

Supplementary Figure S1. Representative images of the spheroids on the plate wells. A – Spheroid from the control (C) condition on the 14th day, at 18 °C; B – Spheroid from control (C) condition on the 14th day, at 23 °C. C – Spheroid exposed to 100 nM of BkF (BkF100) on the 14th day, at 18 °C; D – Spheroid exposed to 100 nM of BkF (BkF100) on the 14th day, at 23 °C.

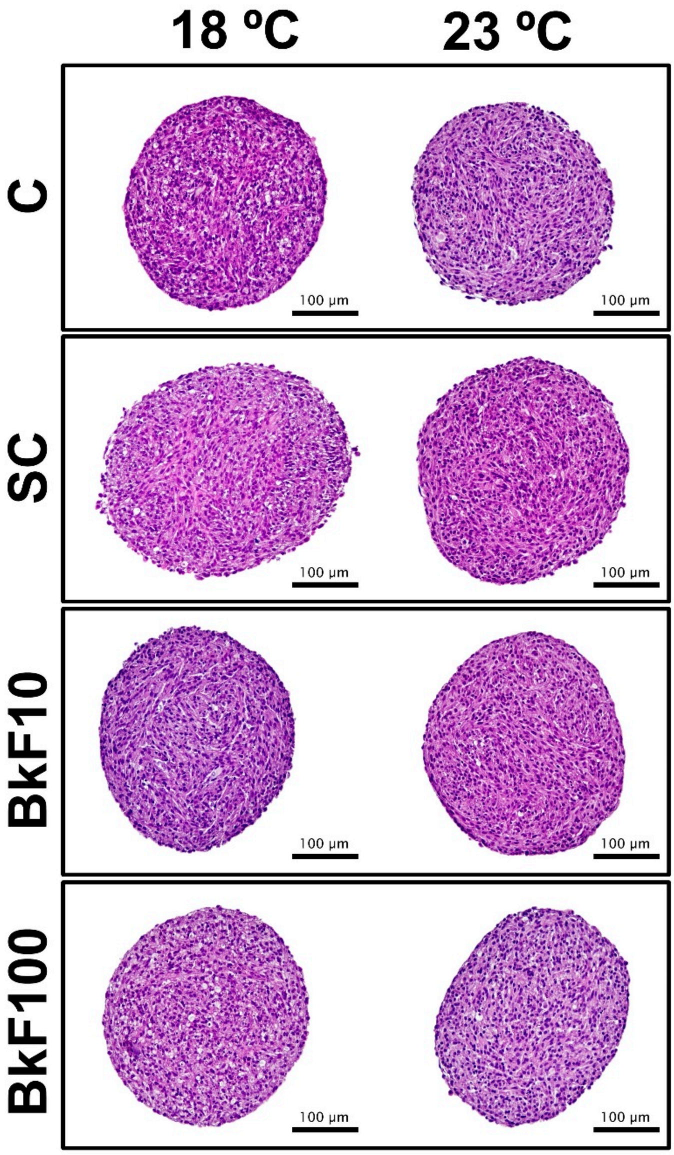

Supplementary Figure S2. Hematoxylin and eosin-stained histological sections of RTL-W1 spheroids, after a 4-day exposure (from day 10 to day 14 in culture) to benzo(k)fluoranthene at 18 °C and 23 °C. C – Control; SC – Solvent Control; BkF10 – 10 nM of BkF; BkF100 – 100 nM of BkF.

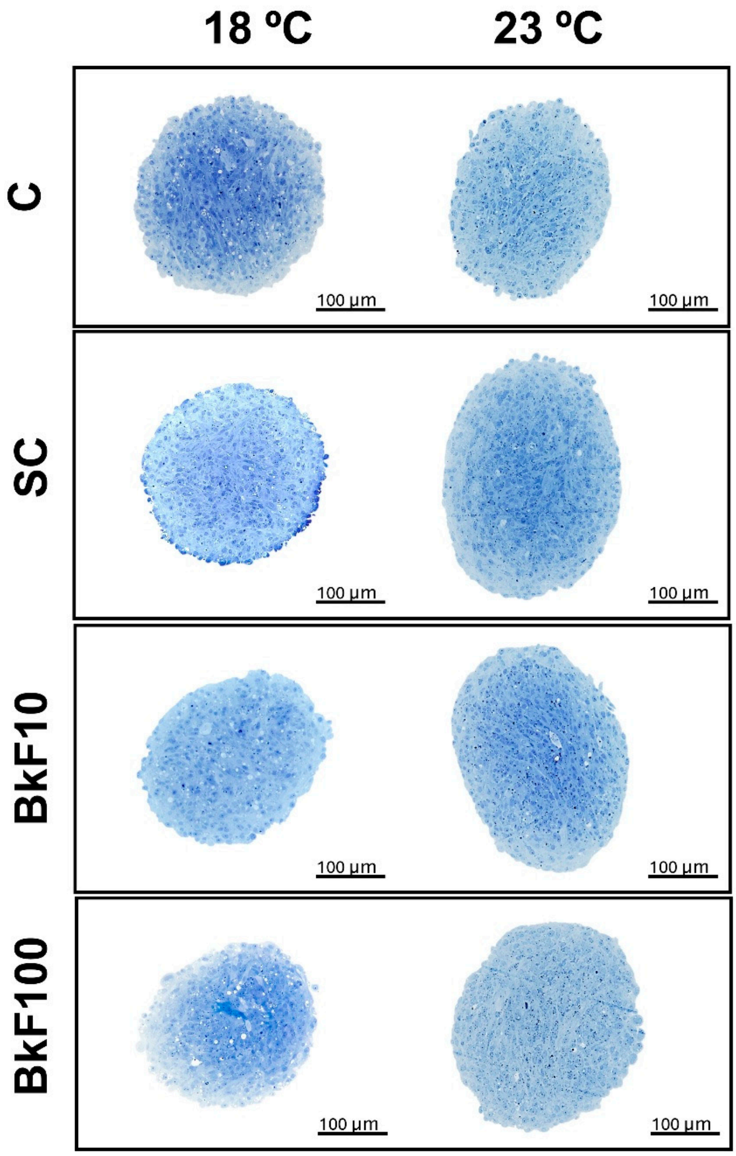

Supplementary Figure S3. Semithin sections of RTL-W1 spheroids, after a 4-day exposure (from day 10 to day 14 in culture) at 18 ºC and 23 °C to Benzo(k)fluoranthene (BkF). C – Control; SC – Solvent Control; BkF10 – 10 nM of BkF; BkF100 – 100 nM of BkF.

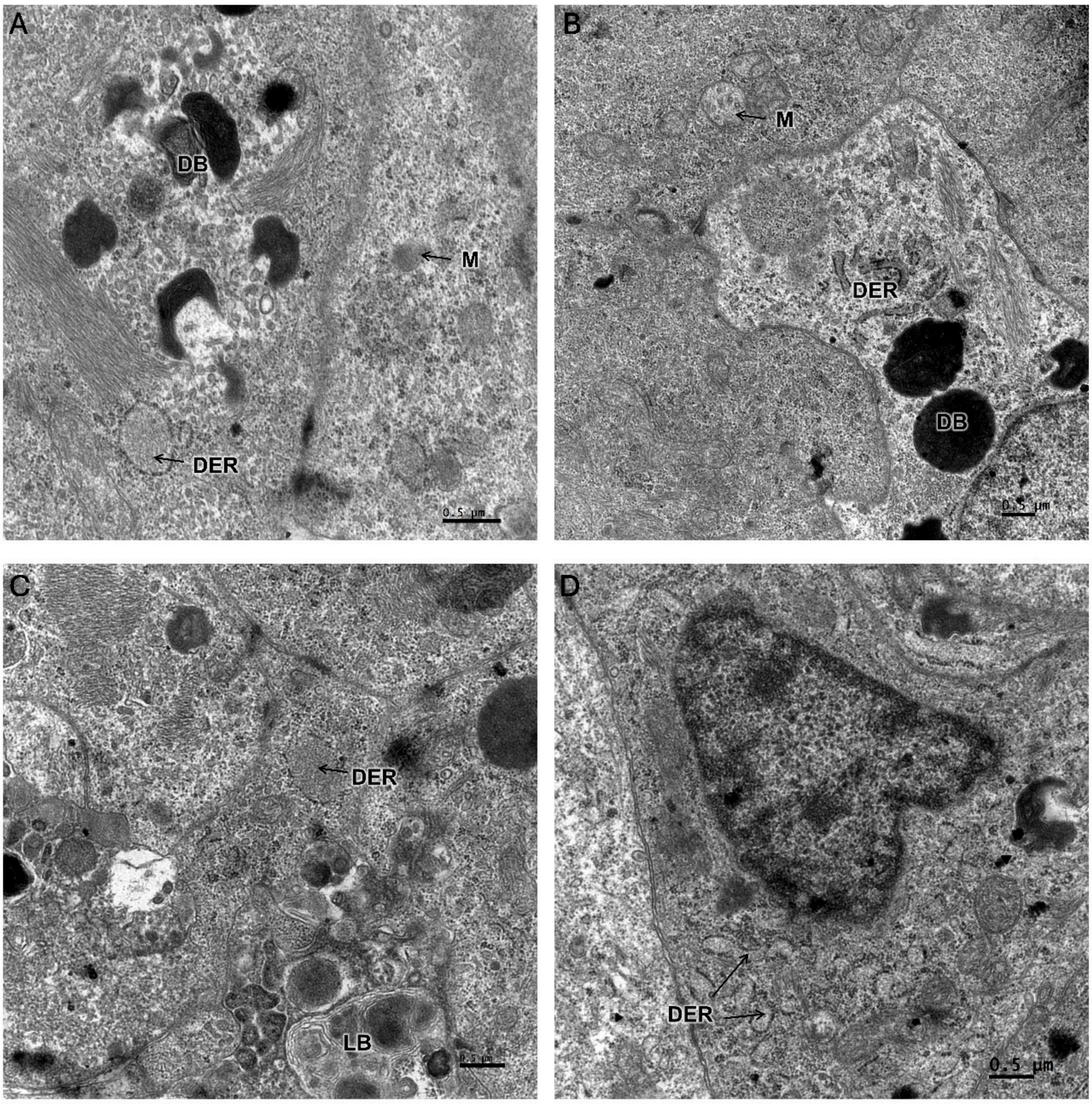

Supplementary Figure S4. Ultrastructural changes in RTL-W1 spheroids (A-D) after a 4-day exposure (from day 10 to day 14 in culture) to 10 nM of benzo(k)fluoranthene (BkF) at 23 °C. DB – Dense bodies accumulation; DER – Dilated endoplasmic reticulum; LB – Lamellar bodies deposition; M – Mitochondria.

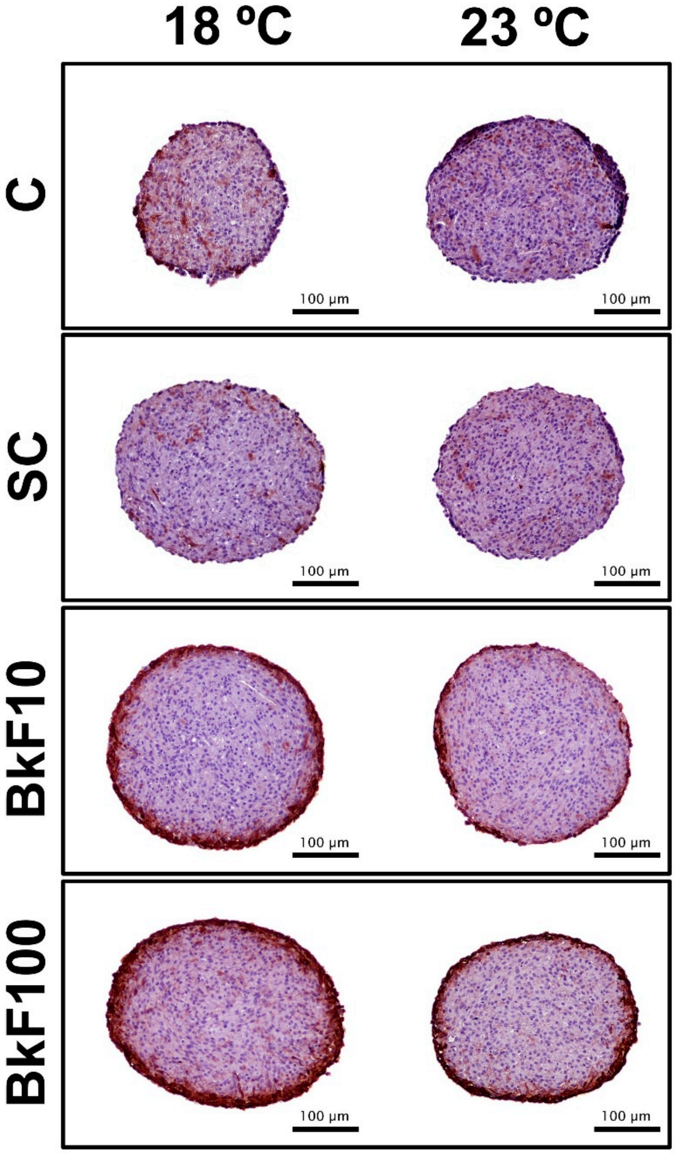

Supplementary Figure S5. CYP1A immunocytochemistry labelling of RTL-W1 spheroids after a 4-day exposure (from day 10 to day 14 in culture) to Benzo(k)fluoranthene at 18 °C and 23 °C. C – Control; SC – Solvent Control; BkF10 – 10 nM of BkF; BkF100 – 100 nM of BkF.

Supplementary Table S1. Frequency of ultrastructural elements of RTL-W1 spheroids after a 4-day exposure (from day 10 to day 14 in culture) to benzo(k)fluoranthene (BkF). Grading scale: 0 (non-observed), 1 (present), 2 (moderately present), and 3 (very frequent). C – Control; SC – Solvent Control; BkF10 – 10 nM of BkF; BkF100 – 100 nM of BkF.

		**C**	**SC**	**BkF10**	**BkF100**
**Endoplasmic Reticulum**	Minimum	3	2	1	1
	Maximum	3	3	3	2
	Median	3	2	1.5	1
	Mode	3	2	1	1
**Golgi Apparatus**	Minimum	0	0	0	0
	Maximum	1	1	0	0
	Median	0.5	0	0	0
	Mode	0	0	0	0
**Lipids**	Minimum	1	1	2	2
	Maximum	3	3	2	2
	Median	1.5	2	2	2
	Mode	1	2	2	2
**Mitochondria**	Minimum	3	3	2	2
	Maximum	3	3	2	2
	Median	3	3	2	2
	Mode	3	3	2	2
**Dense Bodies**	Minimum	3	2	2	1
	Maximum	3	2	3	3
	Median	3	2	3	3
	Mode	3	2	3	3
**Membrane Protrusions**	Minimum	3	2	1	2
	Maximum	3	3	2	3
	Median	3	2	1.5	2
	Mode	3	2	1	2
**Intermediate Filaments**	Minimum	1	2	2	1
	Maximum	3	2	2	2
	Median	2	2	2	1.5
	Mode	2	2	2	1
**Vacuoles**	Minimum	1	1	2	1
	Maximum	2	3	3	3
	Median	1.5	2	2.5	2
	Mode	1	2	3	1
